# Bounded search for de novo identification of degenerate cis-regulatory elements

**DOI:** 10.1186/1471-2105-7-254

**Published:** 2006-05-15

**Authors:** Jonathan M Carlson, Arijit Chakravarty, Radhika S Khetani, Robert H Gross

**Affiliations:** 1 Department of Computer Science and Engineering, University of Washington, Seattle, WA 98105, USA; 2 Department of Cancer Pharmacology, Millennium Pharmaceuticals Inc., Cambridge, MA 02138, USA; 3 Department of Biology, Dartmouth College, Hanover, NH 03755, USA

## Abstract

**Background:**

The identification of statistically overrepresented sequences in the upstream regions of coregulated genes should theoretically permit the identification of potential cis-regulatory elements. However, in practice many cis-regulatory elements are highly degenerate, precluding the use of an exhaustive word-counting strategy for their identification. While numerous methods exist for inferring base distributions using a position weight matrix, recent studies suggest that the independence assumptions inherent in the model, as well as the inability to reach a global optimum, limit this approach.

**Results:**

In this paper, we report PRISM, a degenerate motif finder that leverages the relationship between the statistical significance of a set of binding sites and that of the individual binding sites. PRISM first identifies overrepresented, non-degenerate consensus motifs, then iteratively relaxes each one into a high-scoring degenerate motif. This approach requires no tunable parameters, thereby lending itself to unbiased performance comparisons. We therefore compare PRISM's performance against nine popular motif finders on 28 well-characterized *S. cerevisiae *regulons. PRISM consistently outperforms all other programs. Finally, we use PRISM to predict the binding sites of uncharacterized regulons. Our results support a proposed mechanism of action for the yeast cell-cycle transcription factor Stb1, whose binding site has not been determined experimentally.

**Conclusion:**

The relationship between statistical measures of the binding sites and the set as a whole leads to a simple means of identifying the diverse range of cis-regulatory elements to which a protein binds. This approach leverages the advantages of word-counting, in that position dependencies are implicitly accounted for and local optima are more easily avoided. While we sacrifice guaranteed optimality to prevent the exponential blowup of exhaustive search, we prove that the error is bounded and experimentally show that the performance is superior to other methods. A Java implementation of this algorithm can be downloaded from our web server at .

## Background

Transcriptional responses are modulated by DNA-binding proteins known as transcription factors, which typically bind sets of similar DNA sequences (cis-regulatory elements). Cognate binding sites for a transcription factor exhibit position-specific variation. Critical residues that mediate transcription factor binding are constrained, while the other residues are free to drift neutrally [1], leading to highly degenerate cis-regulatory elements that are often hard to detect computationally.

The *de novo *computational identification of cis-regulatory elements is an extensively studied problem. Numerous motif-finding algorithms have been developed over the years (for reviews see [2,3]).

Nearly all motif-finding algorithms fall into three general classes: pattern-based, profile-based and combinatorial. Each class of algorithm uses a different mathematical model (referred to as a motif model) to represent a set of cis-regulatory elements. Pattern-based methods employ a consensus motif model in which cis-regulatory elements are represented by short words using the IUPAC alphabet. These methods seek to enumerate all possible words in the set of upstream sequences of coregulated genes in order to identify conserved (statistically overrepresented) motifs. Profile-based methods, on the other hand, are based on a Position Weight Matrix motif model. These methods try to identify statistically overrepresented motifs by comparing upstream sequences (for instance, by multiple sequence alignments) and seeking out local similarities. Combinatorial motif-finding programs employ a position-independent mismatch motif model, where the motif is typically represented as a word of length *l *with at most *k *mismatches. These methods seek to identify cis-regulatory elements by clustering closely related groups of words.

Each motif model has its limitations when searching for highly degenerate cis-regulatory elements. Consensus-based algorithms typically struggle with highly degenerate motifs, in part because of their motif model. Exhaustive enumeration of degenerate motifs over the 15-letter IUPAC alphabet causes an explosion of the search space from 4^*l *^to 15^*l *^(for a motif of length *l*) [4]. Further, the limited expressiveness of the consensus model implies that motifs represented via this model are at best crude approximations of the actual cis-regulatory elements. The Position Weight Matrix model is more expressive, but is also prone to local maxima due to the enormous size of its search space. Highly degenerate cis-regulatory elements that fit the position-independent mismatch model are difficult to find in published databases of cis-regulatory elements [5]. All of these models fail to account for inter-position dependencies. Barash *et al. *showed that modeling a collection of binding sites as a mixture of two Position Weight Matrices can account for some positional dependencies [6], while King and Roth present a nonparametric, PWM-based method that can account for arbitrary dependencies [7].

In this paper we present a novel approach to the discovery of highly degenerate cis-regulatory elements that combines aspects of all three motif models. Our approach starts with the most overrepresented non-degenerate words in a set of upstream regions. For each word, we explore the mismatch space immediately surrounding it, generalizing the word to a degenerate consensus that is more significantly overrepresented than the original word. We then construct a Position Weight Matrix based on the actual occurrences of the consensus in the given set of upstream regions. This approach leverages the representational accuracy of the Position Weight Matrix model while reducing the problem of local maxima through discretization. Implicit in the approach is the assumption that a set of binding sites described by an overrepresented degenerate cis-regulatory element has at least one element within it that is itself overrepresented. In this paper, we prove that the statistical significance of a degenerate motif is bounded by the sum of the significance of the non-degenerate motifs it describes. This bound validates our assumptions and provides leverage for efficient identification of degenerate motifs. We demonstrate that our method is effective at finding highly degenerate cis-regulatory elements that are best described using the full IUPAC alphabet in *S. cerevisiae*. When tested on biological datasets, our approach outperforms nine other motif-finding programs based on each of the three motif models described above.

## Results

Statistical overrepresentation is often used in motif-finding programs as a surrogate for biological significance. A natural measurement of overrepresentation is the probability of observing at least *k *occurrences of a motif given its frequency in the genome. We estimate this probability using the Poisson distribution, which is (-log)-transformed and Bonferroni-corrected to yield a *Sig *score similar to the binomial-based Sig score described by [8]. The Sig score is easily extended to degenerate motifs when we consider that such a motif describes a collection of non-degenerate motifs. For this reason, we use the terms *composite motif *and *instantiation *to respectively refer to a degenerate motif and the non-degenerate motifs it describes. As shown in the methods, a useful property of the Sig score is that the Sig score of a composite motif is bounded by the sum of the Sig scores of its instantiations.

### Sig score is bounded

**Figure 1:**
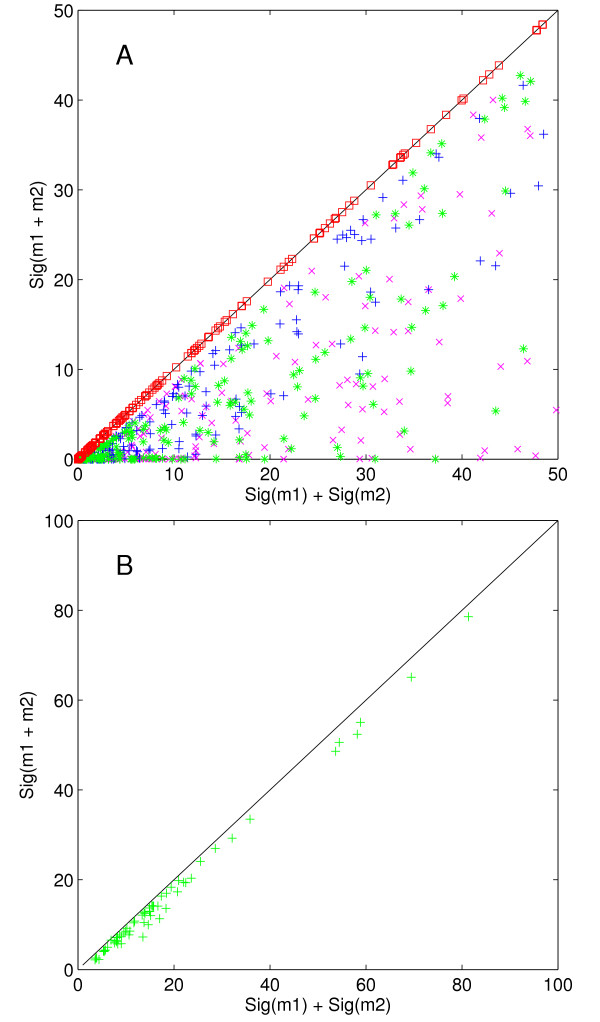
**Experimental demonstration of bounded Sig**. Sig(*m*_1_) + Sig(*m*_2_) is plotted against Sig(*m*_1 _+ *m*_2_), where *m*_1 _and *m*_2 _differ by one mismatch. This assumption assures the existence of a degenerate consensus motif that precisely describes {*m*_1_, *m*_2_}. **A**. Simulation in which *k*, *λ *∈ [0, 50] and *t *= ∞. Four variations were run, in which the parameters for *m*_1 _and *m*_2 _were identical (red □), had different *k *(blue +), had different *λ *(green *) or had different *k *and *λ *(magenta x). **B**. Known binding sites from SCPD that differ by one mismatch. *t *is the number of *l*-length substrings in the 800 bases upstream of the translation start sites of each gene in the regulon.

Previous methods have taken the approach of identifying the highest scoring non-degenerate motifs, then merging those that have a significant amount of textual overlap [8,9]. Implicit in this approach is the assumption that significant degenerate motifs describe a set of significant non-degenerate motifs. To directly investigate the relationship between the Sig scores of two motifs *m*_1 _and *m*_2 _and the Sig score of the composite motif *M *= {*m*_1_, *m*_2_}, we performed the following experiment. We randomly chose values for *k *(the number of occurrences of a motif in a regulon) and *λ *(the expected number of occurrences) for the two motifs and plotted Sig(*k*_1_, *λ*_1_) + Sig(*k*_2_, *λ*_2_) against Sig(*k*_2 _+ *k*_2_, *λ*_1 _+ *λ*_2_). As we demonstrate in Methods, if we consider *m*_1 _and *m*_2 _to be the same motif, the resulting combined motif has expected count *λ*_1 _+ *λ*_2 _and observed count *k*_1 _+ *k*_2 _in the group. This experiment thus investigates the relationship between the Sig scores of two motifs that differ by one mismatch and the Sig score of the degenerate motif that describes exactly those two motifs. When we randomly choose *k *and *λ *from [0, 50] and compute Sig(*k*, *λ*) + Sig(*k*, *λ*), we find that it almost exactly equals Sig(2*k*, 2*λ*) (Figure 1A). When we generalize the experiment to vary *k *and *λ *between the two terms, we find Sig(*k*_1 _+ *k*_2_, *λ*_1 _+ *λ*_2_) ≤ Sig(*k*_1_, *λ*_1_) + Sig(*k*_2_, *λ*_2_). Thus, it appears that Sig(*k*_1_, *λ*_1_) + Sig(*k*_2_, *λ*_2_) bounds Sig(*k*_1 _+ *k*_2_, *λ*_1 _+ *λ*_2_) and that this bound is extremely tight when *k*_1 _≈ *k*_2 _and *λ*_1 _≈ *λ*_2_. This bound also holds when known motifs and regulons taken from the SCPD database [10] that differ by one mismatch are merged (Figure 1B). A proof of this bound is given in Methods.

### Validation of hill climbing algorithm

In order to leverage the bounded Sig in a practical context, we developed an algorithm capable of generalizing the degree of overrepresentation of composite motifs from a single instantiation. This algorithm, referred to as the hill climbing algorithm and described in Methods, attempts to generalize a single non-degenerate motif by exploring the space of motifs that differ by one mismatch from the original motif, and iteratively adding those motifs that maximally improve the Sig score. Thus, given a single, non-degenerate motif *m*, HC(*m*) returns a high-scoring degenerate (composite) motif of any number of instantiations.

In order to validate the hill climbing algorithm in a biological context, we tested the algorithm against the set of *S. cerevisiae *regulons defined in the SCPD database [10]. For each regulon with experimentally verified binding sites, we ran the hill climbing algorithm on each binding site, comparing it with the resulting composite motif via the Φ score metric [11]. The Φ score of a motif measures the nucleotide-level overlap between the sites in a regulon that a motif matches and the SCPD reported binding sites. If the hill climbing algorithm improved the description of the binding sites, this would be reflected in an increase in the Φ scores.

**Figure 2:**
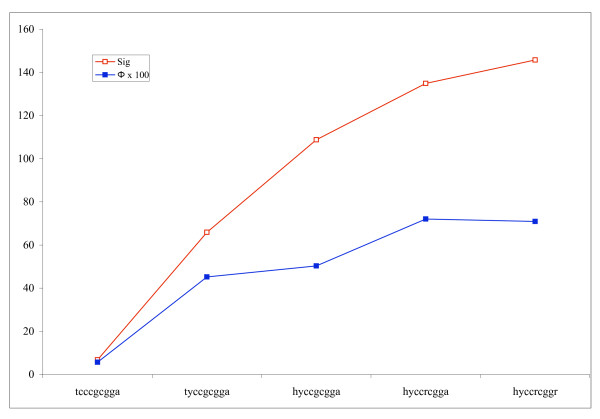
**A trace of the hill climbing algorithm is shown for a binding site of PDR3**. The algorithm goes through 5 iterations, after which no modification further improves the Sig score. The top line traces the Sig score and the bottom line traces Φ × 100.

Table 1 shows the results of running the hill climbing algorithm on all reported binding sites for a number of yeast regulons from the SCPD for which there was more than 1 reported binding site (without a spacer region). Comparing the Φ scores before and after running the hill climbing algorithm compares the ability of the best non-degenerate and degenerate exemplars to model the entire set of binding sites. The motif that gives the best Φ score among hill climbing results for each regulon is displayed as well. As indicated by the increase in average Φ scores, the hill climbing algorithm successfully generalizes many of the non-degenerate binding sites to improve the degree of overlap between the published sites and the predicted motif. The best Φ score improves in 11 out of 16 cases where the Φ score changes more than 0.10 as a result of the hill climbing algorithm. The fact that the Φ score drops in some instances highlights the limitation of Sig as an estimate of biological significance. The details of one run on a binding site of PDR3 are shown in Figure 2 to demonstrate the changes a motif undergoes during the hill climbing algorithm. As can be seen, the hill climbing algorithm steadily improves the Sig score of the motif over multiple iterations. Although the Φ score is not available to the algorithm while it is optimizing the motif, we have included it in this figure to show the relationship between the degree of overrepresentation and the biological relevance of this particular motif.

### Identifying motifs ab initio

As shown in the previous section, given a single non-degenerate instantiation of a biologically relevant motif, the hill climbing algorithm is capable of returning a composite motif that is a more accurate descriptor of the experimentally determined binding sites. Of course, in practical motif-finding situations, this non-degenerate instantiation is not available to the algorithm. However, Theorem 1 states that a composite motif with *n *instantiations and Sig score *σ *must include at least one instantiation with Sig score at least *σ*/*N*. Thus, if we are searching for a highly overrepresented degenerate motif, it is reasonable to start with a list of overrepresented, non-degenerate motifs, each of which is used as a seed from which to begin our search.

We therefore performed the following experiment to determine the practical benefit of the hill climbing algorithm in the *ab initio *discovery of degenerate motifs of variable length. We identified candidate motifs for each regulon, using the oligo-analysis tool from the Regulatory Sequence Analysis Tools website (hereafter referred to as RSAT) [8,12]. RSAT was set to identify the 50 most significant hexamers, then to assemble textually related hexamers into longer motifs. Each motif reported by RSAT was run separately on the hill climbing algorithm, resulting in a set of degenerate motifs. All motifs reported by RSAT were scored for their overlap with the biologically relevant list of binding sites via the Φ score metric. These Φ scores were compared against the Φ scores obtained from the motifs generated by the hill climbing algorithm (columns (c) and (d) in Table 2). After Sinha and Tompa [13], the Φ score for a regulon is computed by sorting the reported motifs by Sig, then reporting the highest Φ score from the top 3 motifs.

The top 3 motifs are examined to account for the possibility that unknown binding sites may exist in these sequences. Surprisingly, while HC(·) improves the performance of RSAT on two regulons, on average, the output of HC decreased the average Φ score by 9% (Table 2). One possible explanation for this is that RSAT finds small fragments of binding sites that, when generalized, include a high number of false positives. This is supported by the observation that the average ΔΦ for those regulons in which the highest scoring motif is a hexamer is -0.11, while the average ΔΦ for those regulons in which the highest scoring motif is longer than 6 bases is +0.5. This may be due to the method RSAT uses to identify motifs longer than 6 bases. In order for such a motif to be reported, a strong linearity assumption is made: both the 6-base prefix and the 6-base suffix must be among the most overrepresented hexamers. Conversely, if any two overrepresented hexamers overlap textually, they will be combined to yield a long motif, regardless of the degree of overrepresentation of the longer motif. This assumption is likely to be violated in practice. To address this, we developed a motif finder specifically aimed at non-degenerate motifs. This algorithm, called BEAM, employs a linearity assumption that is broadly similar to the bound used by the hill climbing algorithm to aggressively limit the search space of motifs. Using a bounded breadth-first (beam) search, BEAM first enumerates all motifs of length 5, then iteratively expands each motif in either direction by concatenating a single nucleotide at either end. In each iteration, BEAM computes the Sig score of each motif, then expands only the highest scoring motifs. The linearity assumptions and error bounds for the BEAM algorithm were previously explored, and the algorithm was shown to be efficient with bounded error [14]. In brief, iteratively expanding motifs works because overrepresented strings are likely to contain overrepresented substrings. Thus, the space of all unambiguous motifs can be efficiently searched by iteratively expanding the most overrepresented motifs. The 50 highest scoring motifs returned by BEAM were used as input to the HC(·) algorithm.

When run alone, BEAM performs comparably to RSAT on these regulons. However, computing HC(·) on each motif reported by BEAM yields a 15% improvement over RSAT (Table 2). For ease of discussion, we named the program that results in running the hill climbing algorithm on the results of BEAM *PRISM *(**P**attern **R**elaxation-based **I**terative **S**earch for **M**otifs). Comparing PRISM and RSAT directly on each regulon reveals that PRISM clearly outperforms RSAT on 6 of 7 regulons where one program outperforms the other by at least 0.10 (referred to as a *clear win*, see [13,15]).

To place PRISM's performance in context, we ran PRISM, RSAT, and eight other popular motif finders on all 28 *S. cerevisiae *regulons from the SCPD for which binding sites have been experimentally characterized (excluding the binding sites for the Zn(II)2Cys6 family of transcription factors, which bind DNA as homodimers with gapped cores). All programs were run from their web servers with default values selected. Where the option was offered, an *S. cerevisiae *background model was specified. PRISM has no adjustable parameters, and hence was not specifically optimized for performance on this dataset. The results are summarized in Table 3 in the format used in Sinha and Tompa [13]. On average, PRISM scores higher than all other programs and, with RSAT and YMF, has the highest number of regulons for which a recognizable portion of the binding sites were discovered (Φ ≥ 0.10). In head-to-head comparisons between PRISM and the other programs, PRISM had 81% of clear wins. PRISM has near-complete descriptions of more cis-regulatory elements than any other program, as measured by the number of regulons for which PRISM's Φ score was at least 0.50.

### Evaluating PRISM in the presence of noise

Input sequences for motif-finding programs typically consist of a set of coordinately expressed genes derived from microarray data. Parallel regulation and rapid serial response of downstream genes are both capable of generating coordinated expression patterns in the absence of coordinate regulation. In addition, errors in clustering may lead to the inclusion of extraneous upstream sequences.

**Figure 3:**
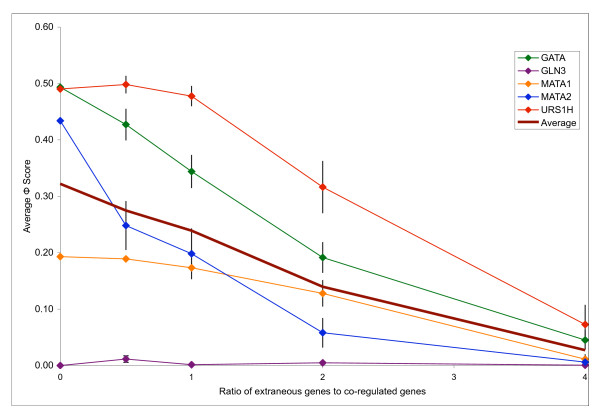
**Corruption tests for PRISM on five randomly selected regulons**. Averages and standard errors over 10 trials are plotted.

To test the robustness of PRISM to increasing levels of extraneous upstream sequences in the dataset, we performed the following experiment on five randomly selected regulons. For each regulon, we added a number of randomly selected upstream regions, corresponding to 0.5, 1, 2 and 4 times the number of sequences in the original regulon. We ran PRISM on each of these data points and assessed its accuracy using the Φ score metric. The results are summarized in Figure 3. As can be seen, PRISM is robust in the presence of extraneous genes. In the presence of twice as many extraneous upstream sequences as real ones, the average Φ decreases from 0.32 to 0.18. PRISM's robustness in the face of noisy gene sets makes it a practical solution for motif-finding from microarray experiments.

As a separate test, we looked at PRISM's performance in the presence of randomly selected gene sets with no coregulated genes present. To test this, randomly selected genes were assembled into regulons of size 3, 5, 10 and 20 (4000 regulons in total). PRISM was run on these regulons, and the Sig score of the top-scoring motif was reported. The results were compared to the Sig scores obtained on the SCPD regulons. The mean Sig score from the random regulons was 12, while the mean Sig score of the SCPD regulons was 22. 58% of the SCPD regulons and 10% of the randomly generated regulons had a Sig score of 20 or above, (for Sig scores of 30 or above, these numbers were 43% and 2% respectively).

### Creating sequence logos from degenerate consensus motifs

**Figure 4:**
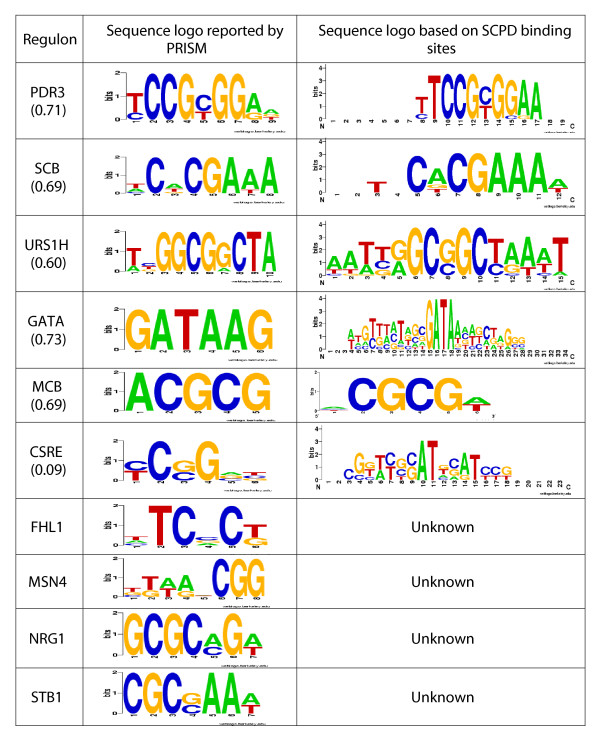
**Sequence logos of motifs predicted by PRISM**. Number next to regulon indicates Φ score of this sequence logo against the SCPD binding sites. Logos generated by WebLogo [38].

Our results suggest that, for *S. cerevisiae *regulons, using the consensus model to arrive at degenerate motifs is more accurate than using a Position Weight Matrix for the same purpose. Of the motif-finding programs tested, AlignAce, MotifSampler, BioProspector, MEME and CONSENSUS all use Position Weight Matrices, and all were outperformed by all three consensus programs (PRISM, RSAT and YMF). These results are broadly consistent with other performance comparisons [13,16]. However, the representational power of the final output may be improved somewhat by converting the consensus sequences into position weight matrices based on the sequences in the regulon that match the consensus. Figure 4 compares the predicted sequence logos from 6 regulons (the 5 highest scoring regulons and a representative low scoring regulon) to the sequence logos from the binding sites as reported by SCPD. We also generated predictions for four transcription factors whose binding sites have not yet been experimentally characterized. The regulons for these transcription factors were identified from data generated using Chromatin Immunoprecipitation (ChIP) [17]. We selected regulons where all regulator-gene interactions had a p-value of at most 0.001 in the ChIP analysis and were confirmed by gene-specific PCR.

### Using PRISM to generate hypotheses

We now show that PRISM is capable of generating directly testable mechanistic hypotheses. As an example, it is known that the transition from G1 to S-phase during the yeast cell cycle is dependent on two heterodimeric complexes, Sbf (Scb-binding factor) and Mbf (Mcb-binding factor). These complexes have the same regulatory subunit Swi6, but have unique DNA-binding proteins, Swi4 and Mbp1 respectively [18]. These two proteins share 50% identity in their DNA-binding domains, and there is a 31% overlap between the *in vivo *targets of Mbf and Sbf, as determined by ChIP data [19]. However, at the nucleotide level, the Φ score for the SCB and MCB cis-regulatory elements is only 0.09. Clearly, the functional overlap between these genes is greater than the overlap at the nucleotide level of their binding sites.

One possible explanation for this apparent contradiction is the involvement of a third protein. A likely candidate for mediating the functional overlap is Stb1, a Swi6-associated protein that has been shown to be involved in the transcriptional regulation of G1 to S-phase transition [20]. In order to address the potential role of Stb1 in Mbf- and Sbf-mediated transcription, we used PRISM to look for overrepresented motifs upstream of genes that were identified as being bound by Stb1 [17]. The cis-regulatory element identified by PRISM (Figure 4) for Stb1 overlaps the Mbf binding sites with a Φ score of 0.17, and overlaps Sbf binding site with a Φ score of 0.20. Thus, it is possible that Stbl mediates the functional overlap between Mbf and Sbf. The association of Stb1 with Swi6, a common subunit of both Sbf and Mbf, supports this hypothesis.

## Discussion

We have shown that the degree of statistical overrepresentation of a degenerate motif is bounded by the sum of the degree of overrepresentation of its non-degenerate instantiations. This deceptively simple relationship sets a lower bound on the Sig score of the most overrepresented instantiation of a highly degenerate motif, since overrepresented motifs will possess one or more instantiations that are themselves overrepresented, albeit to a lesser degree. PRISM leverages this bound to provide a rapid, effective means of identifying highly degenerate overrepresented motifs, starting from non-degenerate motifs. While we have demonstrated PRISM on co-regulated sets of genes, it is relatively straight forward to apply the bound for phylogenetic footprinting, an orthogonal approach to motif finding that leverages the evolutionary conservation of transcription factor binding sites [21-23].

Comparing the performance of PRISM to motif finders based on other motif models reveals a consistent pattern. On this dataset, using the Φ score metric, PRISM outperforms PWM-based motif finding programs (AlignACE, BioProspector, MEME, MotifSampler, MITRA and Consensus) by large margins, ranging from 50 to 200%. On the other hand, the difference between PRISM and programs based on word counting (YMF, Weeder, RSAT) is much smaller, ranging from 15 to 30%. In general, motif finders perform best on synthetic data generated according to their motif model [13,24]. Thus, this observation provides some hints at the mechanism of the underlying DNA-protein interaction: if the free energy of binding of a protein to a cis-regulatory element is very tightly correlated with the additive overrepresentation of each position, programs based on word counting would be expected to perform poorly compared to programs based on the more flexible PWM-based search, which emphasizes position independence. This is because PWM search algorithms can directly optimize the contributions of individual bases even in the absence of overrepresented words that connect adjacent positions. Thus, PRISM's superior performance relative to PWM-based programs may be indicative of the limitations of the additivity assumption inherent in PWMs and their optimization algorithms. These limitations are demonstrated by experimental results that suggest that the simple additivity assumption in protein-DNA binding encoded in the PWM is little more than a first approximation [25-27]. A number of groups have extracted common principles to identify underlying mechanisms in protein-DNA recognition from the solved structures of protein-DNA cocrystals, which contain hundreds of examples of binding contacts. These analyses have demonstrated that interactions often occur between multiple DNA bases and amino acid side chains. For instance, the widespread Zif268-like zinc finger transcription factors consist of three domains, each of which recognizes overlapping trinucleotides [28,29]. In this case the protein-DNA contacts are clearly neither position independent, nor additive. A broad study of protein-DNA binding contacts has shown that, although the chemical properties of the base-amino acid contacts might be additive and position-independent in some cases, the same is not true for the stereochemical effects of adjacent residues on a DNA double helix [30].

Not surprisingly, models of protein-DNA binding that include position dependent effects more accurately model the true binding sites of a transcription factor than do simple PWMs, though at the expense of more free parameters [6,7,31]. Our solution to this problem is to use the PWM representation in the final output, but to restrict the independence assumption by using a word-based search strategy that assumes the presence of overrepresented instantiations. The experimental results validate this decision. There is a second potential explanation for the performance difference between PWM-based programs and consensus-based programs on this dataset. PWMs search a much larger parameter space than consensus models, as each position has three free parameters (the probability of three of the four bases), each of which is a real number between 0 and 1. In contrast, every position in a consensus model is represented with one parameter, which can take on one of 15 values. Thus, employing a Position Weight Matrix representation during the motif search frames the problem in more complex terms than employing a consensus representation. This added complexity leads to search strategies that are more prone to local maxima and that often over fit to include noise when learning novel cis-regulatory elements that occur only a handful of times in the group. For instance, Gibbs Sampler (originally developed to search for highly degenerate motifs) is very sensitive to noise. The dilution of ten target sequences containing a planted motif with five random sequences reduces the accuracy of Gibbs Sampler from 90% to 30% [32]. MotifSampler, a derivative of Gibbs Sampler, does not perform well on the SCPD datasets.

Although PRISM outperforms the other programs by a substantial margin on this randomly selected dataset from *S. cerevisiae *(winning in 81% of the cases where there is a clear difference), it is formally possible that a performance comparison on other datasets will lead to a different result. Since motif finders are classifiers, the "no free lunch" theorem of machine learning states that, for any given motif finder, there must exist a dataset for which that motif finder outperforms PRISM [33]. However, such datasets that are also biologically relevant may be rare, as evidenced by the small number of clear losses. Since our test set was fairly large (constituting about 50 to 60% of all published *S. cerevisiae *regulons), we believe that the results reported here will generalize to most yeast regulons. We wish to emphasize that the dataset (and the criteria) presented here have been used in previous published performance comparisons [13,15]. It is also interesting to note that our comparison of ten motif-finding programs on 28 regulons is larger than any previously published comparison of motif finders except Tompa *et al. *[16], which takes a philosophically different approach.

We wish to place special emphasis on the fact that our algorithm has no nuisance parameters. This is significant as it makes PRISM robust to use by non-experts and enables blind testing to be performed in a fair way on regulons with known binding sites. It is circular to attempt to tune the parameters of motif-finding programs in tests involving *ab initio *motif discovery, unless the tuning is performed on a separate training set of data. To our knowledge, there has been no publication to date involving motif-finding programs with tunable parameters that demonstrates error rates on separate training and test sets. In some cases, performance comparisons have been performed with methods tuned for optimal performance on each individual regulon, clearly violating notions of circularity and overfitting [15]. Thus, the generalizability of tunable parameters in the field of motif-finding remains an open question. We have assumed that the web-based interfaces of the other programs tested here contain reasonable parameter estimates for a naive user interested in motif finding. The fact that PRISM has no tunable parameters enables a fair comparison to be made. However, we acknowledge that it may be possible for expert users running the other programs to obtain higher scores than were obtained here. Such improved performance would best be demonstrated in a rigorous resampling framework.

The chief limitation of the algorithm presented here is that it is not likely to work for cis-regulatory elements that contain widely spaced critical residues. An example of this is the Zn(II)2Cys6 binuclear cluster transcription factor family in yeast and other fungi (see [34] for review). The binding sites of members of this family consist of two sets of critical residues separated by a long spacer region. We are developing a separate algorithmic approach to this problem that leverages the bound shown here (Chakravarty *et al*., in preparation).

Finally, the bound stated in Theorem 1 holds for all (-log)-transformed probability distributions. While overrepresentation has proved to be a useful approximation to biological significance, it clearly has its limitations, as evidenced by the regulons for which all the tested motif finders failed to find a plausible match. By dramatically reducing the search space, we anticipate that PRISM, like its predecessor BEAM [14], will be able to leverage complex statistical measures that more closely approximate biological significance. We are currently exploring such metrics.

## Conclusion

We have shown that the statistical overrepresentation of a collection of binding sites is bounded by the sum of the overrepresentation of each distinct sequence. This bound lead to a simple hill climbing algorithm, which we showed outperforms a wide variety of commonly used motif finding programs. PRISM's success highlights the limitations of assuming independence between nucleotide positions and supports the growing body of evidence that protein-DNA binding is best modeled when dependencies are considered. In this light, PRISM's main contribution is the demonstration that a simple linear approach can account for potential dependencies and can identify a likely set of binding sites from which more descriptive models can be inferred. While the PRISM method is limited in its generality, as it cannot handle gapped binding sites, the theorem proved here is guiding the algorithmic development of a program that identifies such binding sites.

## Methods

### Notation

We define a composite motif *M *= {*m*_1_, *m*_2_,..., *m*_*n*_} to be a set of *n *= |*M*| non-degenerate motifs, each of length *l*. We refer to each *m*_*i *_∈ *M *as an instantiation of *M*. Degenerate consensus motifs over the IUPAC alphabet are special cases of composite motifs for which *n *= 2^*a*^3^*b *^for some non-negative integers *a *and *b *and each motif *m*_*i *_∈ *M *differs from *m*_*i*+1 _by exactly one mismatch. For such motifs, an equivalent notation is given by *M *= *b*_1_*b*_2_...*b*_*n*_, where *b*_*i *_is an IUPAC symbol describing a subset of A, C, G, T. We write |*b*_*i*_| to mean the size of that subset. For example, WAS = {AAC, AAG, TAG, TAG}, and |*b*_1_| = |*b*_3_| = 2, |*b*_2_| = 1.

### Overrepresentation

PRISM is given a set *S *= {*s*_1_, *s*_2_,...,*s*_*c*_} of DNA sequences of lengths *t*_1_, *t*_2_,..., *t*_*c*_, and seeks to return the most overrepresented composite motif *M *in *S*. In this section, we first define overrepresentation for a single motif with respect to *S*, then generalize to composite motifs. We demonstrate that the overrepresentation of a composite motif is bounded by the the sum of the overrepresentation of its instantiations.

#### Single motifs

Let *X *be a non-negative, integer-valued random variable that describes the number of times motif *m *occurs in *S*. Overrepresentation of a motif *m *that occurs *X *= *k *times in *S *is given by

Pr{*X *≥ *k *| S
 MathType@MTEF@5@5@+=feaafiart1ev1aaatCvAUfKttLearuWrP9MDH5MBPbIqV92AaeXatLxBI9gBamrtHrhAL1wy0L2yHvtyaeHbnfgDOvwBHrxAJfwnaebbnrfifHhDYfgasaacH8akY=wiFfYdH8Gipec8Eeeu0xXdbba9frFj0=OqFfea0dXdd9vqai=hGuQ8kuc9pgc9s8qqaq=dirpe0xb9q8qiLsFr0=vr0=vr0dc8meaabaqaciaacaGaaeqabaWaaeGaeaaakeaaimaacqWFse=uaaa@3845@},     (1)

where *S *is drawn from some distribution S
 MathType@MTEF@5@5@+=feaafiart1ev1aaatCvAUfKttLearuWrP9MDH5MBPbIqV92AaeXatLxBI9gBamrtHrhAL1wy0L2yHvtyaeHbnfgDOvwBHrxAJfwnaebbnrfifHhDYfgasaacH8akY=wiFfYdH8Gipec8Eeeu0xXdbba9frFj0=OqFfea0dXdd9vqai=hGuQ8kuc9pgc9s8qqaq=dirpe0xb9q8qiLsFr0=vr0=vr0dc8meaabaqaciaacaGaaeqabaWaaeGaeaaakeaaimaacqWFse=uaaa@3845@. To compute this probability, we assume that each *s*_*i *_∈ *S *is generated by some generative model *μ *(or equivalently, the distribution of *X *is given by *μ*). For concreteness, we take *μ*, to be an *l *- 1 order Markov model (see Implementation section below), though in general other distributions are feasible. For simplicity, we further assume that the t=∑i=1c(ti−l+1)
 MathType@MTEF@5@5@+=feaafiart1ev1aaatCvAUfKttLearuWrP9MDH5MBPbIqV92AaeXatLxBI9gBaebbnrfifHhDYfgasaacH8akY=wiFfYdH8Gipec8Eeeu0xXdbba9frFj0=OqFfea0dXdd9vqai=hGuQ8kuc9pgc9s8qqaq=dirpe0xb9q8qiLsFr0=vr0=vr0dc8meaabaqaciaacaGaaeqabaqabeGadaaakeaacqWG0baDcqGH9aqpdaaeWaqaaiabcIcaOiabdsha0naaBaaaleaacqWGPbqAaeqaaOGaeyOeI0IaemiBaWMaey4kaSIaeGymaeJaeiykaKcaleaacqWGPbqAcqGH9aqpcqaIXaqmaeaacqWGJbWya0GaeyyeIuoaaaa@3E99@ motifs of length *l *generated by *μ *are independent trials. While this simplification ignores the fact that the motif at each position will depend on the *l *- 1 previous motifs and will affect the *l *- 1 following ones, it allows us to compute Probability (1) using a hyper-geometric derived distribution. Previous studies have found that the effects of this independence assumption are negligible except for highly repetitive motifs [8]. Our implementation of BEAM masks out such motifs as a preprocessing step.

While the hyper-geometric distribution is the most accurate model given our assumptions, it is generally too computationally expensive to be of practical use in this context. Instead, an approximation using the binomial [8,9] or Gaussian [4] has previously been used. We have chosen the Poisson distribution, as it efficiently and accurately approximates the binomial distribution when the number of trials *t *is large and the probability *p*_*m *_that any given trial results in *m *is small.

The Poisson distribution parameterizes *X *by *λ *= E[*X*] and approximates Probability (1) as

Pr⁡{X≥k|λ}=∑j=ktλje−λj!.     (2)
 MathType@MTEF@5@5@+=feaafiart1ev1aaatCvAUfKttLearuWrP9MDH5MBPbIqV92AaeXatLxBI9gBaebbnrfifHhDYfgasaacH8akY=wiFfYdH8Gipec8Eeeu0xXdbba9frFj0=OqFfea0dXdd9vqai=hGuQ8kuc9pgc9s8qqaq=dirpe0xb9q8qiLsFr0=vr0=vr0dc8meaabaqaciaacaGaaeqabaqabeGadaaakeaacyGGqbaucqGGYbGCcqGG7bWEcqWGybawcqGHLjYScqWGRbWAcqGG8baFiiGacqWF7oaBcqGG9bqFcqGH9aqpdaaeWbqaamaalaaabaGae83UdW2aaWbaaSqabeaacqWGQbGAaaGccqWGLbqzdaahaaWcbeqaaiabgkHiTiab=T7aSbaaaOqaaiabdQgaQjabcgcaHaaaaSqaaiabdQgaQjabg2da9iabdUgaRbqaaiabdsha0bqdcqGHris5aOGaeiOla4IaaCzcaiaaxMaadaqadaqaaiabikdaYaGaayjkaiaawMcaaaaa@50A3@

If *λ *≪ *t *then we can approximate Equation (2) by summing to infinity, which gives us the standard tail probability. We expect binding sites to be rare, so we will use this latter definition.

#### Composite motifs

Now consider the general case of composite motifs. A composite motif can be viewed as an equivalence class over its instantiations. That is, the composite motif *M *describes a set of binding sites and we are interested in the overrepresentation of those motifs as a set, not individually.

**Observation 1. ***Let X*_1_, *X*_2_,..., *X*_*n *_*be independent, Poisson distributed random variables with expectations λ*_1_, *λ*_2_,...,*λ*_*n*_. *Then *X=∑i=1nXi
 MathType@MTEF@5@5@+=feaafiart1ev1aaatCvAUfKttLearuWrP9MDH5MBPbIqV92AaeXatLxBI9gBaebbnrfifHhDYfgasaacH8akY=wiFfYdH8Gipec8Eeeu0xXdbba9frFj0=OqFfea0dXdd9vqai=hGuQ8kuc9pgc9s8qqaq=dirpe0xb9q8qiLsFr0=vr0=vr0dc8meaabaqaciaacaGaaeqabaqabeGadaaakeaacqWGybawcqGH9aqpdaaeWaqaaiabdIfaynaaBaaaleaacqWGPbqAaeqaaaqaaiabdMgaPjabg2da9iabigdaXaqaaiabd6gaUbqdcqGHris5aaaa@3858@*is Poisson distributed with expectation *λ=∑i=1nλi
 MathType@MTEF@5@5@+=feaafiart1ev1aaatCvAUfKttLearuWrP9MDH5MBPbIqV92AaeXatLxBI9gBaebbnrfifHhDYfgasaacH8akY=wiFfYdH8Gipec8Eeeu0xXdbba9frFj0=OqFfea0dXdd9vqai=hGuQ8kuc9pgc9s8qqaq=dirpe0xb9q8qiLsFr0=vr0=vr0dc8meaabaqaciaacaGaaeqabaqabeGadaaakeaaiiGacqWF7oaBcqGH9aqpdaaeWaqaaiab=T7aSnaaBaaaleaacqWGPbqAaeqaaaqaaiabdMgaPjabg2da9iabigdaXaqaaiabd6gaUbqdcqGHris5aaaa@3950@.

*Proof*. The expectation is evident from the interpretation of the parameter *λ *as the expected number of occurrences. That *X *is Poisson distributed can be proved using the generating function of the Poisson distribution [35].□

If we let *X*_*i *_be the number of observed occurrences of *m*_*i *_∈ *M *in *S*, then *X *is the number of observed occurrences of *M *and Observation 1 implies that

Pr⁡{X≥k|λ}=Pr⁡{∑i=1nXi≥k|∑i=1nλi},     (3)
 MathType@MTEF@5@5@+=feaafiart1ev1aaatCvAUfKttLearuWrP9MDH5MBPbIqV92AaeXatLxBI9gBaebbnrfifHhDYfgasaacH8akY=wiFfYdH8Gipec8Eeeu0xXdbba9frFj0=OqFfea0dXdd9vqai=hGuQ8kuc9pgc9s8qqaq=dirpe0xb9q8qiLsFr0=vr0=vr0dc8meaabaqaciaacaGaaeqabaqabeGadaaakeaacyGGqbaucqGGYbGCcqGG7bWEcqWGybawcqGHLjYScqWGRbWAcqGG8baFiiGacqWF7oaBcqGG9bqFcqGH9aqpcyGGqbaucqGGYbGCcqGG7bWEdaaeWbqaaiabdIfaynaaBaaaleaacqWGPbqAaeqaaOGaeyyzImRaem4AaSMaeiiFaW3aaabCaeaacqWF7oaBdaWgaaWcbaGaemyAaKgabeaakiabc2ha9bWcbaGaemyAaKMaeyypa0JaeGymaedabaGaemOBa4ganiabggHiLdaaleaacqWGPbqAcqGH9aqpcqaIXaqmaeaacqWGUbGBa0GaeyyeIuoakiabcYcaSiaaxMaacaWLjaWaaeWaaeaacqaIZaWmaiaawIcacaGLPaaaaaa@5DC1@

provided the *X*_*i *_are independent. This independence assumption is equivalent to the assumption for single motifs that each trial is independent from all other trials. As in the single motif case, if *λ *≪ *t*, the primary violation of this assumption will occur for auto-correlated motifs. In the composite motif context, auto-correlation occurs when the last *w *letters of *m*_*i *_are the same as the first *w *letters of *m*_*j*_. In such cases, the statistical significance will be overestimated. Like the single motif case, this tends to be a problem only with highly repetitive motifs, though the odds of *M *collectively describing a highly repetitive degenerate motif increases with *n*.

#### Bounding composite motif significance

A useful property of Equation (3) is its relationship to the significance of each *X*_*i*_. If *k *is the number of times *M *occurs in *S*, we can write *k *= ∑_*i*_*k*_*i*_, where *k*_*i *_is the number of times *m*_*i *_∈ *M *occurs in *S*. In the simple case where *M *= {*m*_1_, *m*_2_}, we can make use of the following general bound.

**Lemma 1. ***Given two independent random variables X and Y*,

Pr{*X *+ *Y *≥ *k*_1 _+ *k*_2_} ≥ Pr{*X *≥ *k*_1_}Pr{*Y *≥ *k*_2_},

*with equality when k*_1 _= *k*_2 _= 0.

*Proof*. By definition

Pr⁡{X≥k1∧Y≥k2}=∑i=k1∞∑j=k2∞Pr⁡{X=i∧Y=j}.
 MathType@MTEF@5@5@+=feaafiart1ev1aaatCvAUfKttLearuWrP9MDH5MBPbIqV92AaeXatLxBI9gBaebbnrfifHhDYfgasaacH8akY=wiFfYdH8Gipec8Eeeu0xXdbba9frFj0=OqFfea0dXdd9vqai=hGuQ8kuc9pgc9s8qqaq=dirpe0xb9q8qiLsFr0=vr0=vr0dc8meaabaqaciaacaGaaeqabaqabeGadaaakeaacyGGqbaucqGGYbGCcqGG7bWEcqWGybawcqGHLjYScqWGRbWAdaWgaaWcbaGaeGymaedabeaakiabgEIizlabdMfazjabgwMiZkabdUgaRnaaBaaaleaacqaIYaGmaeqaaOGaeiyFa0Naeyypa0ZaaabCaeaadaaeWbqaaiGbccfaqjabckhaYjabcUha7jabdIfayjabg2da9iabdMgaPjabgEIizlabdMfazjabg2da9iabdQgaQjabc2ha9bWcbaGaemOAaOMaeyypa0Jaem4AaS2aaSbaaWqaaiabikdaYaqabaaaleaacqGHEisPa0GaeyyeIuoaaSqaaiabdMgaPjabg2da9iabdUgaRnaaBaaameaacqaIXaqmaeqaaaWcbaGaeyOhIukaniabggHiLdGccqGGUaGlaaa@60A6@

**Figure 5:**
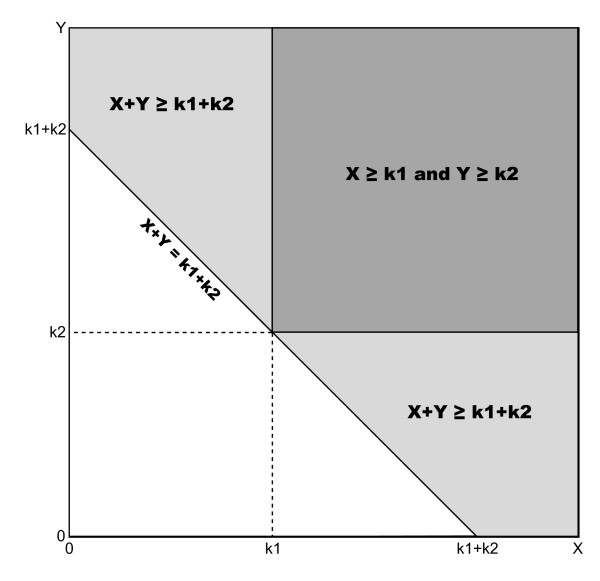
**Probability space of *X *≥ *k*_1 _∧ *Y *≥ *k*_2 _is a subset of *X *+ *Y *≥ *k*_1 _+ *k*_2_**. *P*(*X *≥ *k*_1 _∧ *Y *≥ *k*_2_) is the sum of the probabilities taken over all values in the dark gray space, while *P*(*X *+ *Y *≥ *k*_1 _+ *k*_2_) is the sum of the probabilities taken over both the light gray and dark gray spaces.

The domain of the summation is shown as the dark gray region in Figure 5. We can visualize the event *X *+ *Y *= *k*_1 _+ *k*_2 _as the line *x *+ *y *= *k*_1 _+ *k*_2 _in Figure 5. The event *X *+ *Y *≥ *k*_1 _+ *k*_2 _is then the area above that line, which must be a superset of the space defined by *X *≥ *k*_1 _∧ *Y *≥ *k*_2_. Thus, we have

Pr{*X *+ *Y *≥ *k*_1 _+ *k*_2_}

   ≥ Pr{*X *≥ *k*_1 _∧ *Y *≥ *k*_2_)}

   = Pr{*X *≥ *k*_1_}Pr{*Y *≥ *k*_2_)}.     □

Lemma 1 extends easily to the general case where *M *has *n *instantiations.

**Corollary 1. ***For the independent random variables X*_1_, *X*_2_,..., *X*_*n*_, *the composite variable *X=∑i=1nXi
 MathType@MTEF@5@5@+=feaafiart1ev1aaatCvAUfKttLearuWrP9MDH5MBPbIqV92AaeXatLxBI9gBaebbnrfifHhDYfgasaacH8akY=wiFfYdH8Gipec8Eeeu0xXdbba9frFj0=OqFfea0dXdd9vqai=hGuQ8kuc9pgc9s8qqaq=dirpe0xb9q8qiLsFr0=vr0=vr0dc8meaabaqaciaacaGaaeqabaqabeGadaaakeaacqWGybawcqGH9aqpdaaeWaqaaiabdIfaynaaBaaaleaacqWGPbqAaeqaaaqaaiabdMgaPjabg2da9iabigdaXaqaaiabd6gaUbqdcqGHris5aaaa@3858@, *and *k=∑i=1nki
 MathType@MTEF@5@5@+=feaafiart1ev1aaatCvAUfKttLearuWrP9MDH5MBPbIqV92AaeXatLxBI9gBaebbnrfifHhDYfgasaacH8akY=wiFfYdH8Gipec8Eeeu0xXdbba9frFj0=OqFfea0dXdd9vqai=hGuQ8kuc9pgc9s8qqaq=dirpe0xb9q8qiLsFr0=vr0=vr0dc8meaabaqaciaacaGaaeqabaqabeGadaaakeaacqWGRbWAcqGH9aqpdaaeWaqaaiabdUgaRnaaBaaaleaacqWGPbqAaeqaaaqaaiabdMgaPjabg2da9iabigdaXaqaaiabd6gaUbqdcqGHris5aaaa@38A4@,

Pr⁡{X≥k}≥∏i=1nPr⁡{Xi≥ki}
 MathType@MTEF@5@5@+=feaafiart1ev1aaatCvAUfKttLearuWrP9MDH5MBPbIqV92AaeXatLxBI9gBaebbnrfifHhDYfgasaacH8akY=wiFfYdH8Gipec8Eeeu0xXdbba9frFj0=OqFfea0dXdd9vqai=hGuQ8kuc9pgc9s8qqaq=dirpe0xb9q8qiLsFr0=vr0=vr0dc8meaabaqaciaacaGaaeqabaqabeGadaaakeaacyGGqbaucqGGYbGCcqGG7bWEcqWGybawcqGHLjYScqWGRbWAcqGG9bqFcqGHLjYSdaqeWbqaaiGbccfaqjabckhaYjabcUha7jabdIfaynaaBaaaleaacqWGPbqAaeqaaaqaaiabdMgaPjabg2da9iabigdaXaqaaiabd6gaUbqdcqGHpis1aOGaeyyzImRaem4AaS2aaSbaaSqaaiabdMgaPbqabaGccqGG9bqFaaa@4C58@.

### Sig score

Throughout a run of PRISM, *S *remains fixed. It is therefore convenient to write the significance of *M *with respect to *S *as a function in *M *that increases monotonically with *M*'s statistical significance as defined by Equation (1). We therefore define

Sig_*S*_(*M*) = -log(Pr{*M *| S
 MathType@MTEF@5@5@+=feaafiart1ev1aaatCvAUfKttLearuWrP9MDH5MBPbIqV92AaeXatLxBI9gBamrtHrhAL1wy0L2yHvtyaeHbnfgDOvwBHrxAJfwnaebbnrfifHhDYfgasaacH8akY=wiFfYdH8Gipec8Eeeu0xXdbba9frFj0=OqFfea0dXdd9vqai=hGuQ8kuc9pgc9s8qqaq=dirpe0xb9q8qiLsFr0=vr0=vr0dc8meaabaqaciaacaGaaeqabaWaaeGaeaaakeaaimaacqWFse=uaaa@3845@}).     (4)

When the specific sequence set is not of interest, we simply write Sig(*M*). Corollary 1 easily maps into the Sig domain.

**Lemma 2. ***Given the composite motif M *= {*m*_1_, *m*_2_,...,*m*_*n*_},

Sig(M)≤∑i=1nSig(mi).     (5)
 MathType@MTEF@5@5@+=feaafiart1ev1aaatCvAUfKttLearuWrP9MDH5MBPbIqV92AaeXatLxBI9gBaebbnrfifHhDYfgasaacH8akY=wiFfYdH8Gipec8Eeeu0xXdbba9frFj0=OqFfea0dXdd9vqai=hGuQ8kuc9pgc9s8qqaq=dirpe0xb9q8qiLsFr0=vr0=vr0dc8meaabaqaciaacaGaaeqabaqabeGadaaakeaacqqGtbWucqqGPbqAcqqGNbWzcqGGOaakcqWGnbqtcqGGPaqkcqGHKjYOdaaeWbqaaiabbofatjabbMgaPjabbEgaNjabcIcaOiabd2gaTnaaBaaaleaacqWGPbqAaeqaaOGaeiykaKcaleaacqWGPbqAcqGH9aqpcqaIXaqmaeaacqWGUbGBa0GaeyyeIuoakiabc6caUiaaxMaacaWLjaWaaeWaaeaacqaI1aqnaiaawIcacaGLPaaaaaa@493D@

*Proof*. The statement is evident from Corollary 1 and the rules of logarithms.     □

Thus, we have

**Theorem 1. ***Given the composite motif M *= {*m*_1_, *m*_2_,...,*m*_*n*_)}, *if *Sig(*M*) = *σ*, *then there exists m*_*i *_∈ *M such that *Sig(*m*_*i*_) ≥ *σ*/*n*.

*Proof*. From Lemma 2 it is evident that the average Sig score of the instantiations is at least *σ*/*n*. So by the Fubini principle, at least one of the instantiations has a Sig score of at least *σ*/*n*.     □

Returning to Figure 5, consider the case where *X *and *Y *are independent. Then the joint probability is given by multiplying the probabilities. If *X *and *Y *have the same distribution parameters, then a three dimensional plot over the probability space of Figure 5 would yield maximum values along the line *y *= *x*. Since independently changing *k*_1 _and *k*_2 _moves the origin of the dark gray region along the line *x *+ *y *= *k*_1 _+ *k*_2_, *P*(*X *≥ *k*_1_)*P*(*Y *≥ *k*_2_) will be most similar to *P*(*Z *≥ *k*_1 _+ *k*_2_) when *k*_1 _= *k*_2_. If the distribution parameters of *X *and *Y *are not equal, the maximum values may not lie along the *y *= *x *line, so *P*(*X *≥ *k*_1_)*P*(*Y *≥ *k*_2_) may not be as good an approximation of *P*(*Z *≥ *k*_1 _+ *k*_2_). Translated into -log space, these observations explain the results seen in Figure 1A.

### Multiple hypothesis correction

BEAM identifies non-degenerate motifs by heuristically searching the entire space of motifs. Since the number of motifs of a given length increases as 4^*l*^, we are more likely to discover high scoring motifs of long lengths simply by chance. To correct for this artifact of multiple hypothesis testing, we applied a Bonferroni correction to Sig to penalize long motifs. The corrected Sig is defined to be

Sig_*S*_(*M*) = -log(Pr{*M *| S
 MathType@MTEF@5@5@+=feaafiart1ev1aaatCvAUfKttLearuWrP9MDH5MBPbIqV92AaeXatLxBI9gBamrtHrhAL1wy0L2yHvtyaeHbnfgDOvwBHrxAJfwnaebbnrfifHhDYfgasaacH8akY=wiFfYdH8Gipec8Eeeu0xXdbba9frFj0=OqFfea0dXdd9vqai=hGuQ8kuc9pgc9s8qqaq=dirpe0xb9q8qiLsFr0=vr0=vr0dc8meaabaqaciaacaGaaeqabaWaaeGaeaaakeaaimaacqWFse=uaaa@3845@} × *f*(*M*)),     (6)

where *f*(*M*) is the number of motifs considered in the process of selecting *M*. Since this number if difficult to determine, we used the approximation of [8], which defines *f*(*M*) to be the number of possible motifs of length *l*. In the non-degenerate case, this yields *f*(*M*) = 4^*l*^. In the degenerate case, we generalize *f*(*M*) to be the number of possible motifs of the same length and degeneracy of *M*:

f(M)=∏i=1l(4|bi|).     (7)
 MathType@MTEF@5@5@+=feaafiart1ev1aaatCvAUfKttLearuWrP9MDH5MBPbIqV92AaeXatLxBI9gBaebbnrfifHhDYfgasaacH8akY=wiFfYdH8Gipec8Eeeu0xXdbba9frFj0=OqFfea0dXdd9vqai=hGuQ8kuc9pgc9s8qqaq=dirpe0xb9q8qiLsFr0=vr0=vr0dc8meaabaqaciaacaGaaeqabaqabeGadaaakeaacqWGMbGzcqGGOaakcqWGnbqtcqGGPaqkcqGH9aqpdaqeWbqaamaabmaabaqbaeqabiqaaaqaaiabisda0aqaaiabcYha8jabdkgaInaaBaaaleaacqWGPbqAaeqaaOGaeiiFaWhaaaGaayjkaiaawMcaaaWcbaGaemyAaKMaeyypa0JaeGymaedabaGaemiBaWganiabg+GivdGccqGGUaGlcaWLjaGaaCzcamaabmaabaGaeG4naCdacaGLOaGaayzkaaaaaa@45E0@

While adding this definition of *f*(*M*) to Sig means the inequality of Lemma 2 will not always hold, running PRISM on the 28 SCPD regulons with a definition of *f*(*M*) that maintains Lemma 2 yields the same average Φ score as using Equation (6) (data not shown). We chose Equation (6) because it conforms to the intuitive notion that a motif should not be penalized if wildcard N characters are appended to it. It should also be noted that this definition only applies to degenerate consensus motifs, and not generalized composite motifs.

### PRISM

#### Leveraging a bounded Sig

As defined above, a degenerate consensus motif is a special case of composite motif wherein each instantiation *m*_*i *_∈ *M *differs from *m*_*i*+1 _by exactly one mismatch. Theorem 1 implies that the search for the most overrepresented degenerate motifs can start with the most overrepresented non-degenerate motifs, provided the number of instantiations is not too large with respect to the level of overrepresentation. Lemma 2 says that Sig({*m*_1_, *m*_2_} ≤ Sig(*m*_1_) + Sig(*m*_2_). While maximizing Sig(*m*_2_) does not imply that Sig({*m*_1_, *m*_2_} will be maximized, Figure 1 suggests that the bound tends to be tight if *m*_1 _and *m*_2 _are similar. Thus, defining

*m*_*i*+1 _= argmax_*m'*_Sig(*m'*),     (8)

where argmax_*x*_*f*(*x*) returns that argument *x *which maximizes *f*(*x*), is a reasonable heuristic. Following our definition of a degenerate consensus motif, the domain of *m*' is constrained to be those motifs that differ from *m*_*i *_by one mismatch.

#### Algorithm

Equation (8) can be implemented as a simple hill climbing algorithm that starts from a single non-degenerate motif and builds a progressively more degenerate motif by iteratively adding those closely-related motifs that most improve the Sig score. Given *M *= {*m*_1_, *m*_2_,...,*m*_*n*_}, let *M*_*i,j *_= {*m*_*i*_, *m*_*i*+1_,...,*m*_*j*_} for 1 ≤ *i *≤ *j *≤ *n*. We can then define the recurrence

*M*_*i*,*j*+1 _= *M*_*i*,*j *_∪ {argmax_*m'*_Sig(*M*_*i*,*j *_∪ {*m'*})}     (9)

over all motifs *m*' that differ from *m*_*j *_by one mismatch (excluding, of course, *m*_*j*-1_). Note that the maximization is taken over *M*_*i,j *_∪ {*m*'} rather than *m*'. While computationally more expensive, this definition lessens the effect that a loose upper bound will have on the recurrence.

As *M*_*i,j *_grows to include more motifs, λMi,j
 MathType@MTEF@5@5@+=feaafiart1ev1aaatCvAUfKttLearuWrP9MDH5MBPbIqV92AaeXatLxBI9gBaebbnrfifHhDYfgasaacH8akY=wiFfYdH8Gipec8Eeeu0xXdbba9frFj0=OqFfea0dXdd9vqai=hGuQ8kuc9pgc9s8qqaq=dirpe0xb9q8qiLsFr0=vr0=vr0dc8meaabaqaciaacaGaaeqabaqabeGadaaakeaaiiGacqWF7oaBdaWgaaWcbaGaemyta00aaSbaaWqaaiabdMgaPjabcYcaSiabdQgaQbqabaaaleqaaaaa@3386@ and kMi,j
 MathType@MTEF@5@5@+=feaafiart1ev1aaatCvAUfKttLearuWrP9MDH5MBPbIqV92AaeXatLxBI9gBaebbnrfifHhDYfgasaacH8akY=wiFfYdH8Gipec8Eeeu0xXdbba9frFj0=OqFfea0dXdd9vqai=hGuQ8kuc9pgc9s8qqaq=dirpe0xb9q8qiLsFr0=vr0=vr0dc8meaabaqaciaacaGaaeqabaqabeGadaaakeaacqWGRbWAdaWgaaWcbaGaemyta00aaSbaaWqaaiabdMgaPjabcYcaSiabdQgaQbqabaaaleqaaaaa@332A@ will continue to grow. As they do so, they will continue to diverge from the *k *and *λ *of any non-degenerate motif that is a candidate for addition to *M*_*i,j*_. As discussed above, this behavior essentially loosens the bound of Lemma 2 to the point where it is possible that Sig(*M*_*i,j*+1_) ≤ Sig(*M*_*i,j*_). In fact, if we start with a motif that occurs more often than expected (*k *> *λ*), then this condition must occur at least before the composite motif includes every possible instantiation of that length, as such a motif would have the property *k *= *λ *= *t*. This provides a natural stopping condition for the recurrence described by Equation (9).

We can now describe a hill climbing algorithm for the identification of *M *= {*m*_1_, *m*_2_,...,*m*_*n*_} given *m*_*i *_as follows.

**HC **(*m*_1_)

   *M *← *m*_1_

   **while **max_*m' *_Sig(*M *∪ {*m*'}) > Sig(*M*) **do**

      *m*' ← argmax_*m' *_Sig(*M *∪ {*m*'})

      *M *← *M *∪ {*m*'}

   **return ***M*

In practice, the binding sites in a regulon of a transcription factor do not necessarily form the complete set of instantiations. That is, it is often the case where two binding sites differ by more than one mismatch, and the intermediate instantiation is not present in the regulon. It is therefore convenient to use the degenerate consensus motif representation of *M*. In this notation, we can implement argmax_*m' *_Sig(*M *∪ {*m*'}) by iterating over all *b*_*i *_to find the IUPAC symbol b′i
 MathType@MTEF@5@5@+=feaafiart1ev1aaatCvAUfKttLearuWrP9MDH5MBPbIqV92AaeXatLxBI9gBaebbnrfifHhDYfgasaacH8akY=wiFfYdH8Gipec8Eeeu0xXdbba9frFj0=OqFfea0dXdd9vqai=hGuQ8kuc9pgc9s8qqaq=dirpe0xb9q8qiLsFr0=vr0=vr0dc8meaabaqaciaacaGaaeqabaqabeGadaaakeaacuWGIbGygaqbamaaBaaaleaacqWGPbqAaeqaaaaa@2F8C@ ⊇ *b*_*i*_, |b′i
 MathType@MTEF@5@5@+=feaafiart1ev1aaatCvAUfKttLearuWrP9MDH5MBPbIqV92AaeXatLxBI9gBaebbnrfifHhDYfgasaacH8akY=wiFfYdH8Gipec8Eeeu0xXdbba9frFj0=OqFfea0dXdd9vqai=hGuQ8kuc9pgc9s8qqaq=dirpe0xb9q8qiLsFr0=vr0=vr0dc8meaabaqaciaacaGaaeqabaqabeGadaaakeaacuWGIbGygaqbamaaBaaaleaacqWGPbqAaeqaaaaa@2F8C@| ≤ |*b*_*i*_| + 1, such that replacing *b*_*i *_with b′i
 MathType@MTEF@5@5@+=feaafiart1ev1aaatCvAUfKttLearuWrP9MDH5MBPbIqV92AaeXatLxBI9gBaebbnrfifHhDYfgasaacH8akY=wiFfYdH8Gipec8Eeeu0xXdbba9frFj0=OqFfea0dXdd9vqai=hGuQ8kuc9pgc9s8qqaq=dirpe0xb9q8qiLsFr0=vr0=vr0dc8meaabaqaciaacaGaaeqabaqabeGadaaakeaacuWGIbGygaqbamaaBaaaleaacqWGPbqAaeqaaaaa@2F8C@ results in the maximum Sig. For example, if *b*_*i *_= A, then b′i
 MathType@MTEF@5@5@+=feaafiart1ev1aaatCvAUfKttLearuWrP9MDH5MBPbIqV92AaeXatLxBI9gBaebbnrfifHhDYfgasaacH8akY=wiFfYdH8Gipec8Eeeu0xXdbba9frFj0=OqFfea0dXdd9vqai=hGuQ8kuc9pgc9s8qqaq=dirpe0xb9q8qiLsFr0=vr0=vr0dc8meaabaqaciaacaGaaeqabaqabeGadaaakeaacuWGIbGygaqbamaaBaaaleaacqWGPbqAaeqaaaaa@2F8C@ will be one of {A, R, W, M} (the selection of b′i
 MathType@MTEF@5@5@+=feaafiart1ev1aaatCvAUfKttLearuWrP9MDH5MBPbIqV92AaeXatLxBI9gBaebbnrfifHhDYfgasaacH8akY=wiFfYdH8Gipec8Eeeu0xXdbba9frFj0=OqFfea0dXdd9vqai=hGuQ8kuc9pgc9s8qqaq=dirpe0xb9q8qiLsFr0=vr0=vr0dc8meaabaqaciaacaGaaeqabaqabeGadaaakeaacuWGIbGygaqbamaaBaaaleaacqWGPbqAaeqaaaaa@2F8C@ = A indicates no mismatches at this position increase the Sig score). Since the addition of a degenerate base multiplies the number of instantiations by 2, 3 or 4, this method allows the algorithm to effectively skip instantiations that don't exist in the regulon.

The running time of argmax implemented over the IUPAC alphabet is linear in the length of the motif. In the worst case, we will execute the while loop 3*l *times, resulting in a motif consisting of all N's. Thus, the worst case running time of the hill climbing algorithm is *O*(*l*^2^*t*(Sig)), where *t*(Sig) is the time it takes to compute Sig. This approach sacrifices guaranteed optimality for a reduction in running time from *O*(*2*^*l*^) to *O*(*l*^2^). It is particularly relevant to note that this algorithm has no adjustable parameters, and hence does not require optimization.

### Implementation

We implemented the hill climbing algorithm in Java (SDK 1.4). The expected number of occurrences *λ *of a motif *m *is computed using maximum likelihood estimation over the set of sequences corresponding to the 800 base pairs upstream of all reported yeast genes. This computation is facilitated by the use of a suffix array (for review, see [36]), which yields a Sig computation running time on a composite motif *M *of *t*(Sig) = *O*(*ln *log *G*), where *l *and *n *are the length and number of instantiations of *M*, and *G *is the total number of bases in the background sequences. While modest computational gains can be achieved using parameterized models (commonly, low-order Markov models are used to estimate background probabilities), the systemic bias of such models in estimating the background probabilities of cis-regulatory elements justifies the increased complexity required to generate unbiased estimates [14].

Non-degenerate motifs are generated using an implementation of the BEAM algorithm, which returns with high confidence the most overrepresented, non-degenerate motifs of all lengths of at least 5 bases [14].

Sig_*S*_(*M*) can be computed with respect to both strands of *S *by simply including the reverse complements of each *m*_*i *_∈ *M *in *M*. BEAM attaches a boolean flag to each motif indicating whether the reverse complements should be considered. The top motifs reported by BEAM are independently run on HC(·), and the final motifs are sorted by score. If the minimum score and degeneracy (*N*) of target motifs is known *a priori*, we use only those motifs from BEAM that match the Sig threshold given by Theorem 1. In general, this information is not available; thus, we take the top *C *motifs from BEAM. We have found that the top 3 motifs reported by PRISM tend to be invariant for all values of *C *≥ 50.

We refer to the combination of BEAM and the hill climbing algorithm as *PRISM *(Pattern Relaxation-based Iterative Search for Motifs). The average running time of this implementation on the data sets described here was 3.5 seconds on a 3 GHz Intel Pentium 4 processor with 512 MB of RAM. The binary files, documentation and the yeast background sequences are available for download at from the project web site [37].

### Metrics

Given a set of binding sites *B *in the upstream sequences *R *of a regulon, we would like to measure the ability of the hill climbing algorithm to take a single instantiation *b *∈ *B *and generalize it to a composite motif *M *= HC(*b*) that closely approximates the set of all binding sites *B*. To this end, two metrics are employed to compare *b *with HC(*b*). The first metric, ΔSig = Sig(HC(*b*)) - Sig(*b*), quantifies the ability of the hill climbing algorithm to identify more overrepresented (higher scoring) motifs. To quantify the practical similarity between HC(*b*) and the entire set *B *of binding sites, we used a metric defined by Pevzner and Sze [11]. Given two motifs *m*_1 _and *m*_2_, let *I*(*m*_*i*_) describe the actual bases in *R *that are part of a sequence described by *m*_*i*_. The similarity between *m*_1 _and *m*_2 _can then be quantified by

ΦR(m1,m2)=I(m1)∩I(m2)I(m1)∪I(m2).     (10)
 MathType@MTEF@5@5@+=feaafiart1ev1aaatCvAUfKttLearuWrP9MDH5MBPbIqV92AaeXatLxBI9gBaebbnrfifHhDYfgasaacH8akY=wiFfYdH8Gipec8Eeeu0xXdbba9frFj0=OqFfea0dXdd9vqai=hGuQ8kuc9pgc9s8qqaq=dirpe0xb9q8qiLsFr0=vr0=vr0dc8meaabaqaciaacaGaaeqabaqabeGadaaakeaacqqHMoGrdaWgaaWcbaGaemOuaifabeaakiabcIcaOiabd2gaTnaaBaaaleaacqaIXaqmaeqaaOGaeiilaWIaemyBa02aaSbaaSqaaiabikdaYaqabaGccqGGPaqkcqGH9aqpdaWcaaqaaiabdMeajjabcIcaOiabd2gaTnaaBaaaleaacqaIXaqmaeqaaOGaeiykaKIaeyykICSaemysaKKaeiikaGIaemyBa02aaSbaaSqaaiabikdaYaqabaGccqGGPaqkaeaacqWGjbqscqGGOaakcqWGTbqBdaWgaaWcbaGaeGymaedabeaakiabcMcaPiabgQIiilabdMeajjabcIcaOiabd2gaTnaaBaaaleaacqaIYaGmaeqaaOGaeiykaKcaaiabc6caUiaaxMaacaWLjaWaaeWaaeaacqaIXaqmcqaIWaamaiaawIcacaGLPaaaaaa@566E@

Thus, the Φ score is a direct measure of the nucleotide-level overlap between the set of known sites and the set of sites predicted by a motif-finding program.

## Authors' contributions

JMC wrote the proof section, implemented the algorithm, and assisted in the design of the experiments. AC proposed the approach and designed the method and the experiments. RSK wrote the section titled "Using PRISM to generate hypotheses," coordinated the performance comparisons, and helped build the figures. RHG conceived the broader outline of the study, acquired the funding, edited the manuscript, and provided overall guidance. JMC and AC wrote the manuscript. All authors read and approved the final manuscript.

## Figures and Tables

**Table 1:** Performance of the hill climbing algorithm on well-characterized S. cerevisiae regulons. The reported binding sites were each run through the hill climbing algorithm to determine the algorithm's ability to generate a representative consensus motif given only one instantiation. Abbreviations are as follows: |*B*|-the number of genes in the regulon; HC(*b*)-the consensus motif reported by HC(·) that had the highest Φ score; ΔSig-the maximum change in Sig score due to HC for any binding site; Φ *b*-the maximum Φ score for any one binding site; Φ HC-the Φ score after HC is run on the known binding sites.

Regulon	|*B*|	HC(*b*)	ΔSig	Φ *b*	Φ HC
BAS1	13	gagtca	17.80	0.36	0.36
CPF1	3	cwcgtgrm	17.26	0.61	0.55
CSRE	6	tcmwttcayccg	28.33	0.27	0.29
GATA	5	gatwas	40.66	0.73	0.56
GCN4	20	bagtcab	26.10	0.29	0.44
GCR1	9	ctyhc	12.59	0.43	0.18
GLN3	3	ytaatctaatc	10.28	0.63	0.64
HAP2	5	gttggttggtgga	13.37	0.37	0.23
MATA1	3	sactaattaggaaa	10.00	0.33	0.36
MATA2	12	brdgtaadt	29.17	0.21	0.48
MCM1	43	chnwttmggdaa	53.31	0.05	0.29
MCB	4	wcgcg	40.46	0.62	0.78
MIG1	13	ccccrbwww	19.43	0.21	0.38
PDR3	10	hyccrcggr	138.96	0.50	0.71
PHO2	6	gtaaattagttaatt	0.00	0.40	0.21
PHO4	12	cacgtggracta	8.41	0.13	0.14
RAP1	18	acacccagacmkc	12.98	0.09	0.15
REB1	20	bvywacccs	25.01	0.36	0.47
ROX1	8	ycyattgttctc	14.86	0.13	0.38
RPA	3	tctcggcggtta	0.00	0.34	0.34
SCB	8	cdcgawa	37.79	0.58	0.72
SFF	4	aggtmaacaa	5.85	0.25	0.50
STE12	6	atgmaac	24.74	0.48	0.53
TBP	20	yatava	18.44	0.44	0.20
UASCAR	4	ttgccmttmgc	16.63	0.25	0.40
UASH	21	gwgtagtgaca	12.12	0.05	0.05
UASPHR	23	cgtggatgaaac	6.44	0.04	0.04
UIS	5	aaaatagcgcctc	0.00	0.22	0.20
URS1H	12	wdwtwgccsccvw	61.10	0.15	0.65

Average	11		24.21	0.33	0.39

**Table 2:** Performance summary for RSAT, BEAM and PRISM. RSAT and BEAM were each run alone on 15 randomly chosen regulons; the resulting Φ scores are reported in columns 3 and 5, respectively. For each program, we ran HC(·) on the top 50 Sig-scoring motifs from RSAT (column 4) and BEAM (column 6). The combination of BEAM followed by HC is named *PRISM*. To summarize the results, we report the average, the number of regulons each program "wins" (defined to be any regulon for which that program's *φ *score is at least 0.10 and is greater than the other programs), and the number of clear wins and losses PRISM has in a head-to-head comparison against the other program. A clear win (loss) is defined to be a regulon for which PRISM's Φ score is at least 0.10 higher (lower) than the program in question.

Regulon	|*B*|	RSAT	RSAT/HC	BEAM	PRISM
BAS1	13	0.36	0.35	0.28	0.07
GATA	5	0.73	0.56	0.73	0.73
GCN4	20	0.29	0.28	0.50	0.50
GLN3	3	0.00	0.00	0.00	0.00
HAP2	5	0.00	0.03	0.00	0.11
MATA2	12	0.28	0.18	0.19	0.43
MCB	4	0.62	0.55	0.69	0.69
PDR3	10	0.46	0.61	0.50	0.71
REB1	20	0.36	0.02	0.32	0.32
SCB	8	0.52	0.58	0.58	0.69
STE12	6	0.65	0.53	0.57	0.57
UASCAR	4	0.04	0.05	0.04	0.07
UASH	21	0.00	0.02	0.03	0.01
UASPHR	46	0.06	0.11	0.01	0.03
URS1H	12	0.42	0.52	0.52	0.60

Average Φ		0.32	0.29	0.33	0.37
Wins		3.0	1.0	1.5	6.5
clear win for PRISM		6	7	4	
clear loss for PRISM		1	1	1	

**Table 3:** Performance comparison of 10 motif finders on 28 regulons. PRISM and nine popular motif finders where run on the SCPD regulons. The data are summarized as in Table 2. The programs tested were: PRISM, RSAT, Mitra [39], AlignACE [40], MotifSampler (MS) [41], BioProspector (BioProsp) [42], MEME [43], Consensus [44], Weeder [45] and Yeast Motif Finder (YMF) [4]. Some programs did not return values for MCM1, so this regulon was omitted.

	PRISM	RSAT	Mitra	AlignACE	MS	BioProsp	MEME	Consensus	Weeder	YMF
BAS1	0.07	0.36	0.00	0.00	0.04	0.00	0.00	0.00	0.20	0.36
CPF1	0.62	0.00	0.00	0.54	0.00	0.62	0.48	0.00	0.47	0.44
CSRE	0.09	0.25	0.00	0.10	0.00	0.49	0.30	0.00	0.08	0.07
GATA	0.73	0.73	0.21	0.18	0.39	0.13	0.19	0.00	0.29	0.56
GCN4	0.50	0.29	0.02	0.26	0.28	0.00	0.02	0.00	0.37	0.29
GCR1	0.12	0.24	0.18	0.04	0.05	0.10	0.08	0.25	0.08	0.10
GLN3	0.00	0.00	0.02	0.79	0.00	0.00	0.05	0.00	0.00	0.00
HAP2	0.11	0.00	0.02	0.07	0.00	0.18	0.00	0.00	0.01	0.00
MATA1	0.19	0.10	0.26	0.17	0.00	0.49	0.12	0.00	0.06	0.13
MATA2	0.43	0.28	0.08	0.15	0.04	0.24	0.09	0.00	0.25	0.23
MCB	0.69	0.62	0.02	0.40	0.48	0.05	0.26	0.51	0.50	0.64
MIG1	0.01	0.22	0.02	0.14	0.02	0.16	0.21	0.18	0.13	0.29
PDR3	0.71	0.46	0.51	0.76	0.75	0.42	0.37	0.73	0.82	0.74
PHO2	0.01	0.00	0.06	0.08	0.00	0.10	0.00	0.01	0.01	0.00
PHO4	0.25	0.21	0.24	0.23	0.00	0.21	0.33	0.23	0.00	0.18
RAP1	0.18	0.18	0.26	0.02	0.00	0.02	0.01	0.00	0.24	0.18
REB1	0.32	0.36	0.00	0.01	0.47	0.30	0.36	0.00	0.26	0.36
ROX1	0.19	0.33	0.05	0.05	0.00	0.09	0.45	0.00	0.17	0.18
RPA	0.02	0.08	0.00	0.00	0.00	0.05	0.10	0.00	0.00	0.00
SCB	0.69	0.52	0.58	0.08	0.00	0.09	0.40	0.00	0.57	0.50
SFF	0.02	0.00	0.10	0.00	0.00	0.00	0.00	0.00	0.00	0.00
STE12	0.57	0.65	0.08	0.24	0.00	0.00	0.42	0.00	0.37	0.50
TBP	0.00	0.00	0.03	0.00	0.19	0.02	0.02	0.00	0.00	0.01
UASCAR	0.07	0.04	0.11	0.00	0.00	0.10	0.04	0.00	0.02	0.02
UASH	0.01	0.00	0.00	0.00	0.00	0.00	0.00	0.00	0.01	0.00
UASPHR	0.03	0.06	0.04	0.10	0.00	0.03	0.07	0.09	0.10	0.03
UIS	0.16	0.00	0.03	0.00	0.00	0.09	0.02	0.10	0.09	0.01
URS1H	0.60	0.42	0.62	0.00	0.50	0.75	0.41	0.46	0.38	0.40

Average Φ	0.26	0.23	0.13	0.16	0.11	0.17	0.17	0.09	0.20	0.22
Wins	6	1.5	3	2	2	6	2	1	1	1.5
# ≥ 0.50	8	4	3	3	1	2	0	2	3	5
# ≥ 0.33	9	9	3	4	5	5	7	3	7	9
# ≥ 0.10	17	17	10	12	7	14	13	6	14	17

clear win for PRISM		8	11	11	14	9	12	13	10	8
clear loss for PRISM		5	0	2	2	4	3	2	3	2
